# Fecal Microbiota in Premature Infants Prior to Necrotizing Enterocolitis

**DOI:** 10.1371/journal.pone.0020647

**Published:** 2011-06-06

**Authors:** Volker Mai, Christopher Michael Young, Maria Ukhanova, Xiaoyu Wang, Yijun Sun, George Casella, Douglas Theriaque, Nan Li, Renu Sharma, Mark Hudak, Josef Neu

**Affiliations:** 1 Department of Microbiology and Cell Sciences and Emerging Pathogens Institute, University of Florida, Gainesville, Florida, United States of America; 2 Department of Pediatrics, University of Florida, Gainesville, Florida, United States of America; 3 Department of Pediatrics, University of Florida, Jacksonville, Florida, United States of America; 4 Department of Statistics and Genetics Institute, University of Florida, Gainesville, Florida, United States of America; 5 Clinical & Translational Science Institute, University of Florida, Gainesville, Florida, United States of America; Indian Institute of Science, India

## Abstract

Intestinal luminal microbiota likely contribute to the etiology of necrotizing enterocolitis (NEC), a common disease in preterm infants. Microbiota development, a cascade of initial colonization events leading to the establishment of a diverse commensal microbiota, can now be studied in preterm infants using powerful molecular tools. Starting with the first stool and continuing until discharge, weekly stool specimens were collected prospectively from infants with gestational ages ≤32 completed weeks or birth weights≤1250 g. High throughput 16S rRNA sequencing was used to compare the diversity of microbiota and the prevalence of specific bacterial signatures in nine NEC infants and in nine matched controls. After removal of short and low quality reads we retained a total of 110,021 sequences. Microbiota composition differed in the matched samples collected 1 week but not <72 hours prior to NEC diagnosis. We detected a bloom (34% increase) of *Proteobacteria* and a decrease (32%) in *Firmicutes* in NEC cases between the 1 week and <72 hour samples. No significant change was identified in the controls. At both time points, molecular signatures were identified that were increased in NEC cases. One of the bacterial signatures detected more frequently in NEC cases (p<0.01) matched closest to γ*-Proteobacteria*. Although this sequence grouped to the well-studied *Enterobacteriaceae* family, it did not match any sequence in Genbank by more than 97%. Our observations suggest that abnormal patterns of microbiota and potentially a novel pathogen contribute to the etiology of NEC.

## Introduction

Necrotizing enterocolitis (NEC) is a devastating disease with a multi-factorial etiology that affects primarily premature infants and carries a huge burden in terms of mortality, morbidity, and cost to society.[1] One hypothesis suggests that a major etiological factor for NEC is colonization of the neonatal gut with an abnormal microbiota,[2] particularly as NEC typically occurs after days 8–10 d postpartum when anerobic bacteria commonly start to colonize the gut. Furthermore, NEC has not been observed in germfree animals [3] and infants with NEC frequently exhibit concomitant bacteremia and endotoxemia.[4] Although various microbes have been cultured from blood and stools in outbreaks of NEC at single institutions, no single organism has consistently been implicated across centers.[3] The human microbiome project,[5] in conjunction with technologic advances that allow for the molecular identification of a vast array of microbes that are difficult or impossible to culture from the intestine,[6], [7] has given us new tools for generating evidence supporting the “abnormal colonization hypothesis”.[8] Studies have shown that multiple environmental factors affect microbiota development,[9] and others have suggested that modification of the microbiota may confer clinical benefits.[10] Preliminary studies have evaluated fecal microbiota from unaffected preterm infants and from infants with NEC both prior to and during NEC.[11], [12] These studies suggest an association between the pattern of intestinal microbial species and NEC.

In this study, we compared the intestinal microbiota in 9 premature infants with NEC to that of 9 matched and unaffected control infants.

## Materials and Methods

### Patients

Preterm infants were enrolled from three University of Florida affiliated hospitals. We studied infants with birth weights less than or equal to 1,250 grams and gestational ages less than or equal to 32 completed weeks. Infants with known congenital malformations of the intestine such as gastroschisis or atresias, and infants that were not expected to survive beyond the first week, were excluded. All infants who met the inclusion criteria were enrolled in the study shortly after birth. Informed written consent was obtained from a parent by the attending physicians or research nurses at each site. The study was approved by the Institutional Review Boards at the University of Florida.

Nine of the approximately 200 babies enrolled in the study to date met the criteria for diagnosis of NEC (Bell Stage 2 or 3 with radiologic pneumatosis intestinalis and/or portal venous gas; or direct intra-operative confirmation of intestinal pathology). [13], [14] Control infants were selected and matched to NEC case infants using gestational age, birth weight, birth center, date of birth (+/− 2 months), and predominant enteral nutrient (breast milk *vs.*. formula).

### Samples

Beginning with the first stool and continuing until discharge, we collected weekly stool samples from study infants for immediate storage at −80°C. The samples that were selected to be analyzed from cases were those collected one week “prior” (median 7 days, range 3–10 days) to the onset of NEC (and at equivalent time points in controls), and those that were collected most “proximate” to the occurrence of NEC (all were within 72 hours of NEC onset). Samples from control infants were chosen at the same week of life at which the samples from the matched cases were obtained.

### Microbiota Analysis

#### DNA extraction and quality control by denaturing gradient gel electrophoresis (DGGE)

DNA was extracted from 200–300 mg fecal samples using a modified Qiagen stool DNA extraction protocol that included a bead beating step.[15] We used DGGE analysis of the V6–V8 region as described previously,[12] for initial quality control.

#### 16S rRNA sequence analysis

DNA was amplified using a barcoded primer set based on universal primers 27F and 338R as described by Hamady et al.[16] Cleaned PCR products were pooled in equimolar amounts and submitted for sequencing using 454 Flex chemistry. From the resulting raw data set, low quality sequences or sequences with a length less than 100 nucleotides were removed. Sequences were initially analyzed using the Ribosomal Database Project (RDP) pyrosequencing pipeline that included features to calculate diversity indices and rarefaction curves. We then used the ESPRIT algorithm [17] to bin sequences into Operational Taxonomic Units (OTUs) using similarity levels from 99% (species/strain level) to 80% (phylum level). We used the QIIME package [18] to calculate i) Chao rarefaction diversity, which estimates how many OUT's are present in a sample, and ii) UniFrac distances [19] , which allow for a comparison of the distribution of OUT's among samples.

### Statistics

Student's t-test was used when data were normally distributed; otherwise a paired chi square test followed by Fisher combining was used to test for a difference in the prevalence of OTUs. We adjusted for an expected high false discovery rate by increasing the requirement for statistical significance to p<0.01. The QIIME package was used to calculate p-values for differences in UniFrac distances.

## Results

### Patient Characteristics

The mean gestational age of all infants was 26.7 weeks (range, 23 to 30 weeks) and mean birth weight was 960 grams (range, 570 to 1,269 grams), There were 4 females and 5 males in the NEC group and 5 females and 4 males in the control group (Table 1). In each group, four infants were delivered by cesarean section. While all of the cases received multiple antibiotics during the two weeks prior NEC diagnosis, four of the five controls did not receive any antibiotics at the matched period of life. There was no significant differences in predominant diet (breast milk *vs.* formula) in the infants with and without NEC prior to the development of the disease.

**Table 1:** Characteristics of study subjects.

Patient ID	Gender	GAge	Weight	Delivery	Diet	Antibiotics	Fed	Stage	DoL NEC
Case 1	F	29	1020	CS	BM	G,O	1	2	5
Case 2	F	27	1080	SVD	Both	G,M,O	1	2	15
Case 3	M	25	1052	CS	BM	A,G,O	1	2	25
Case 4	M	25	570	SVD	Form	A,G,N,V,Z	6	3	24
Case 5	M	26	967	CS	BM	A,G,N,V	1	3	28
Case 6a	M	23	639	SVD	BM	E,V,G	5	3	11
Case 6b	F	28	1225	SVD	Form	A,G,Z	1	2	5
Case 7	F	27	920	SVD	BM	A,T	2	2	39
Case 8	F	26	750	CS	Both	G,V,Z	6	3	20
Case 9	M	24	734	SVD	BM	G,V,Z	3	3	41
Control 1	M	28	1102	SVD	BM	V	2	X	X
Control 2	M	28	1140	CS	BM	None	5	X	X
Control 3	F	27	875	SVD	BM	C,G	1	X	X
Control 4	F	26	652	SVD	Both	None	2	X	X
Control 5	M	26	1020	CS	BM	A,C,E	11	X	X
Control 6	M	28	1269	CS	BM	A,G	1	X	X
Control 7	F	30	1263	CS	BM	None	1	X	X
Control 8	F	28	1115	SVD	BM	G,N,V	1	X	X
Control 9	F	27	1120	SVD	Both	None	1	X	X

GAge: Gestational age in weeks, Weight: weight at birth in g, Delivery: CS: Caesarian, SVD: Spontaneous Vaginal Delivery, Diet: BM: Breast Milk, Form: Formula, Both: Breast Milk and Formula, O: Oxacillin, G: Gentamicin, M: Meropenem, E: Erythromycin, N: Nafcillin, C: Ceftazidime, T:Tobramycin, Z: Zosyn, Am: Ampicillin, Az: Azithromycin, Fed: day of life at first meal, Stage: Bell's stage, DoL NEC: day of life NEC diagnosed, Case 6a indicates patient before and case 6b patient at diagnosis.

### Microbiota Analysis

After processing and screening of specimens, we analyzed a total of 110,021 sequences that represented an average of 3,056 sequences per sample (range, 1,822–5,625). The average length per sequence was 233 nucleotides (range, 100–292).

We then clustered sequences using ESPRIT at the 98%, 95% and 90% similarity levels to obtain OTUs containing similar sequences for further microbiota analysis. ESPRIT uses a novel alignment algorithm that performs pairwise rather than multiple sequence alignment. In its current implementation it has been shown to improve alignment quality over other commonly used algorithms that include CD-Hit and Uclust. Before removing singletons (OTUs that contain only one sequence) we had 11510, 771 and 350 OTUs at the respective similarity levels. After removal of singletons we retained 7768, 587 and 279 OTUs respectively. To reduce loss of statistical power due to multiple comparisons, we removed all OTUs with less than five sequences. We retained 2636, 365 and 184 OTUs at the respective similarity levels for analysis.

**Figure 1:**
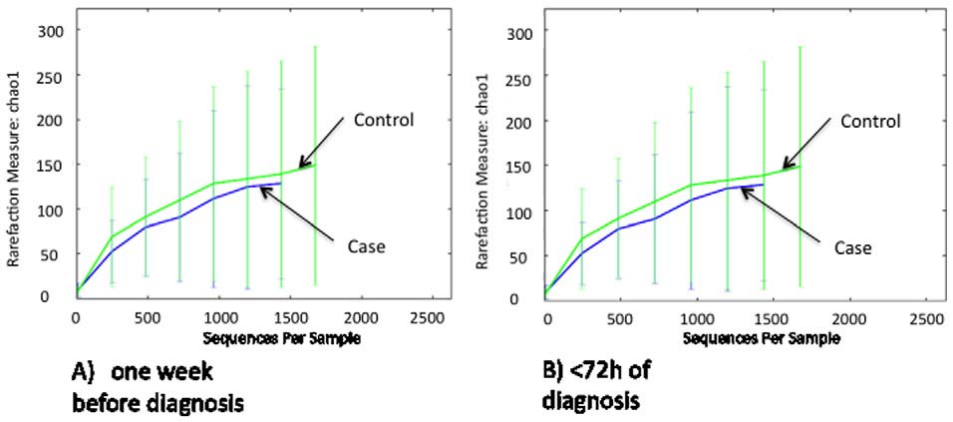
Chao rarefaction diversity. Chao diversity was calculated from sequence distribution A) one week before and B) within 72 hours of NEC diagnosis.

**Figure 2:**
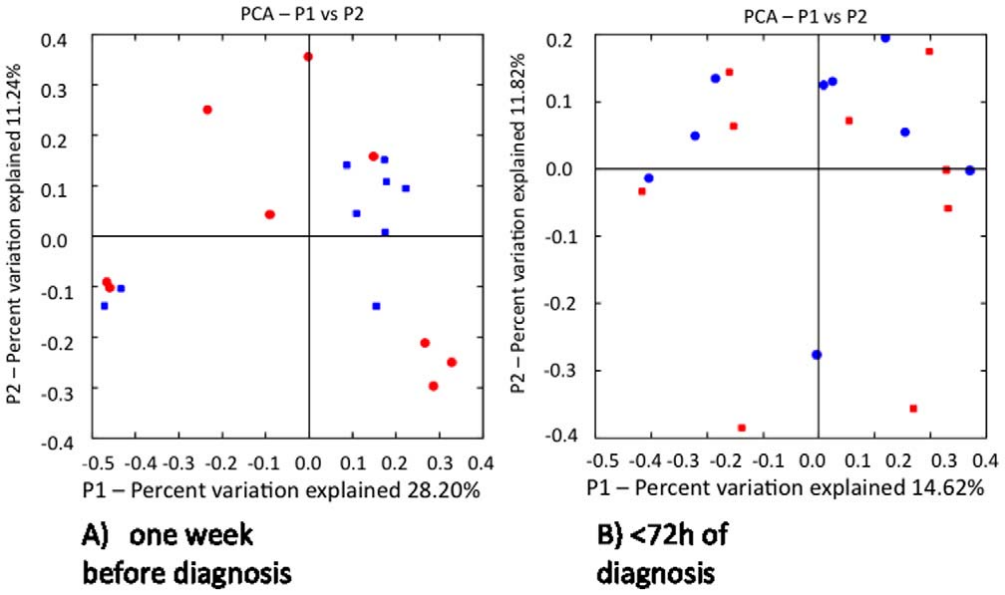
Unifrac diversity measures. Principal component analysis (PCA) of overall diversity based on UniFrac (unweighted) metric A) one week before and B) within 72 hours of NEC diagnosis. Squares represent controls and circles represent cases. P1 is component 1 and P2 component 2.

Using these binned sequence data, we first analyzed microbiota structure and diversity with microbial ecology tools available in the QIIME package. The total numbers of OUT's present, as estimated using Chao1-based rarefaction curves (a common measure of diversity in populations) did not differ between cases and controls at either of the two time points (Figure 1). When we analyzed overall composition using the UniFrac metric (unweighted), which is based on branch length differences between the case/control groups in a phylogenetic tree generated from all 16S rRNA sequences, we detected that one week before NEC diagnosis seven out of the nine control samples clustered closely together, whereas the nine NEC case samples were quite distant from each other (Fig. 2A). We did not observe the same in the samples collected during the week of NEC diagnosis (Fig. 2B). Although diversity indices, which are calculated based on the number and distribution of observed OUT's, did not differ at either time point, there was more variation in overall microbiota structure in NEC infants one week before diagnosis whereas microbiota was more similar in most controls.

**Figure 3:**
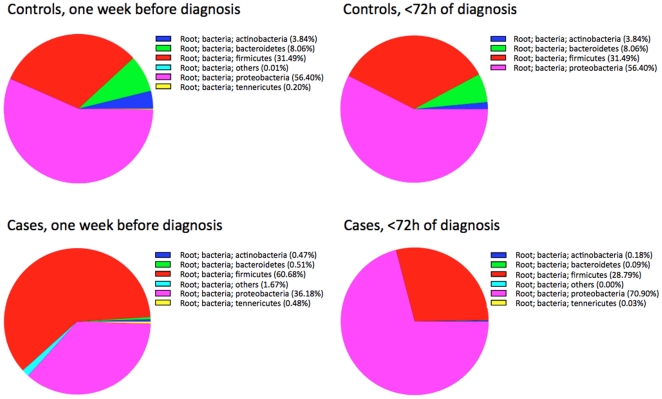
Changes in proportion of bacterial phyla. Proportions of the four major phyla in nine controls and nine cases one week before and within 72 hours of NEC diagnosis.

We next analyzed differences in the proportion of bacterial groups at the phylum level. Four phyla (*Firmicutes, Proteobacteria, Bacteroidetes and Actinobacteria*) dominated the microbiota in most samples (Figure 3). The proportion of *Actinobacteria* and *Bacteroidetes* was lower in NEC than in control infants. We observed a 34% increase in the proportion of *Proteobacteria* in NEC infants at the expense of a 32% decrease in *Firmicutes* that occurred over the week before the diagnosis of NEC. In contrast, the proportions of the four major bacterial phyla in control infants did not change significantly over the same time period. In NEC infants *Firmicutes* dominated at the earlier time point. A late bloom of Palong with continuously low numbers of *Actinobacteria* and *Bacteroidetes* was observed in NEC infants during the week prior to diagnosis of NEC.

**Figure 4:**
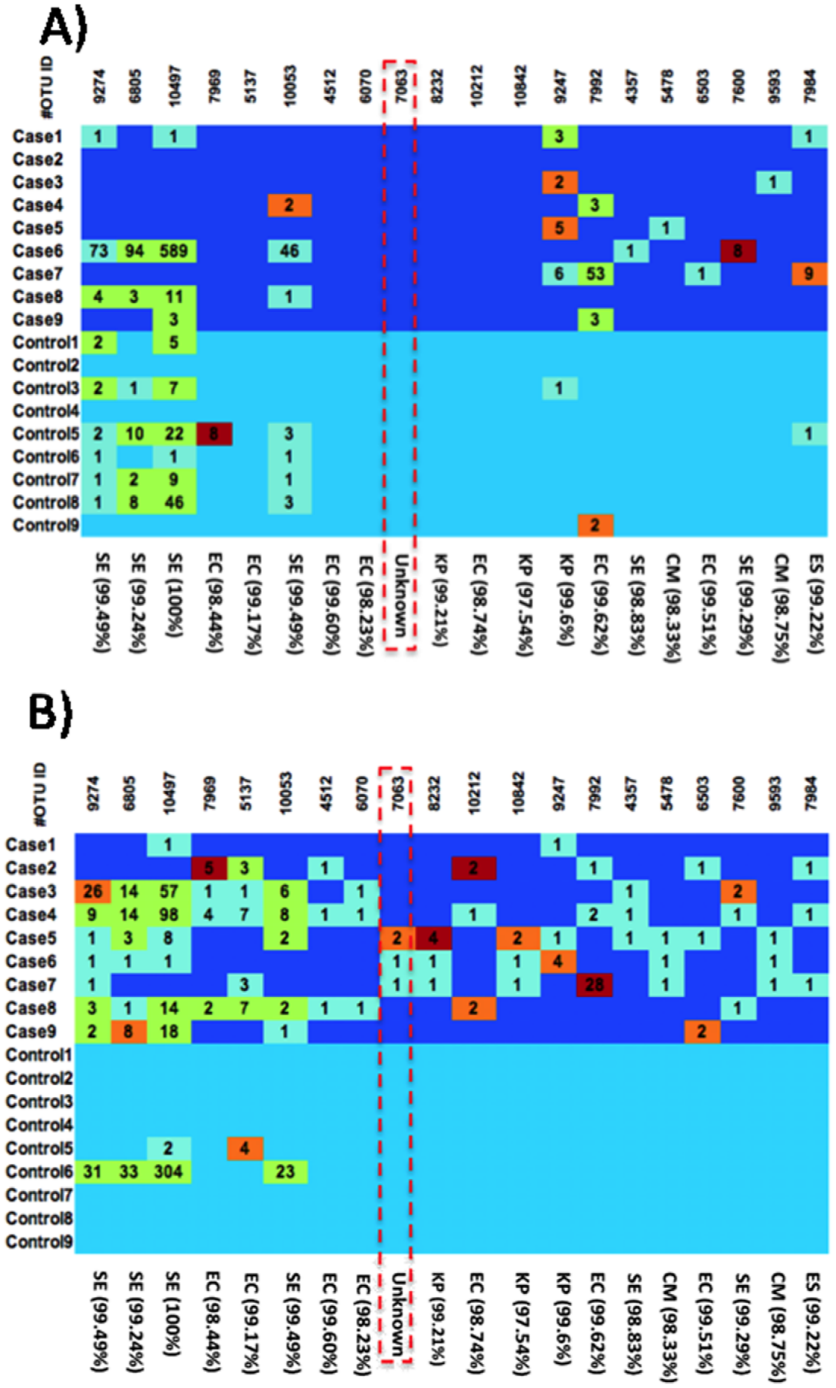
Differences in OTU abundance. Heat map of selected OTUs at 98% similarity by subject A) one week before and B) within 72 hours of NEC diagnosis. Colors indicate the ratio for a particular OTU in a sample to the average ratio of this OTU in all samples. Dark red: >10, light red: >5 and < = 10, light green: >1.5 and < = 5 and light blue: < = 1.5. Numbers indicate how often the OTU was detected. The marked Unknown OTU in B) groups closest to Klebsiella (97%) . CM: *Cronobacter muytjensii*; EC: *Escherichia coli*; ES: *Enterobacter sp. glo8*; KP: *Klebsiella pneumoniae*; SE: *Staphylococcus epidermidis.*

Finally, as an exploratory analysis, we compared the distribution of OTUs between the two groups at both time points. In the hypervariable region of the 16S rRNA that we analyzed, a similarity level of 98% might equate to the level of strain, a similarity level of 95% to the species level, and 90% similarity to the genus level. At the 98% level, a variety of OTUs were more frequently detected (p<0.01) in cases compared to controls (Figure 4). In contrast to other microbiota studies in older children and adults where most of the ‘unknown’ OTUs group to the phylum *Firmicutes* or *Bacteroidetes*, we detected a particular ‘unknown’ OTU that grouped to the *γ-Proteobacteria*. This OTU was detected in 3/9 NEC cases but in none of the controls (p<0.01). This OTU appears most closely related to the *Enterobacteriaceae*, a family that contains many known pathogens and accordingly has been extensively studied. Surprisingly, although the OTU matched this well-studied family, no strains in the database achieved more than a 97% match. In addition to this ‘unknown’ OUT' we detected a variety of other OTUs at the time of diagnosis that were found only in NEC infants. These OTUs grouped closest to known *Enterobacteriaceae* (*Cronobacter muytjensii*, *Escherichia coli*, *Enterobacter sp.*; *Klebsiella pneumoniae*) and to *Staphylococcus epidermidis*.

## Discussion

The healthy adult human harbors roughly 1×10^14^ bacteria in the intestinal tract and these organisms make up about 60% of the total fecal mass.[20] Derangements in the numbers or types of bacteria can affect health and influence disease states. While human data are just starting to emerge, animal studies have suggested that microbiota composition in the neonatal period may affect gastrointestinal (GI) tract development, mucosal integrity, and even nutritional status.[21], [22] Thus, developing an understanding of the neonatal (especially the very preterm) microbiota would be invaluable in helping to elucidate the processes that promote health or alternatively predispose to diseases, such as NEC.

In contrast to an earlier report that suggested reduced microbiota diversity in samples collected from cases after NEC diagnosis and a distinct clustering when compared with matched controls,[11] we did not detect such differences in overall diversity measures in our study. Rather, our findings confirm and extend our earlier observation that overall microbiota diversity at the time of diagnosis does not differ between healthy preterm infants and infants with NEC.[23] Our observation that one week before diagnosis microbial structure was more heterogeneous in infants that developed NEC suggests the potential for early detection of risk and focused intervention. Based on the data presented here it is feasible that future studies that include more cases will allow for the identification of a “high risk” microbiota pattern based on discriminant analysis of 16S rRNA sequences.[24]

We used high throughput sequencing in a prospective study design. Our sequence depth per subject was more than 20 times deeper than that previously reported [11] allowing us to detect rare OTUs that would have been missed with less extensive sequencing. We did confirm the presence of a higher proportion of *Proteobacteria* that the same group reported only after the diagnosis of NEC. [11] Furthermore, we observed that the proportion of *Proteobacteria* increased in samples collected from NEC infants over the week prior to the diagnosis of NEC. In the samples collected from the NEC infants one week before diagnosis, the proportion of *Proteobacteria* was actually lower than that observed in the matched control samples. Hence, our data raise the possibility that preterm infants not sufficiently colonized with *Proteobacteria* during the first weeks of life during which early immune protection or tolerance might develop, may not be able to modulate an adaptive immunological response to a subsequent bloom of *Proteobacteria*, and instead develop intestinal pathology consistent with NEC.

A recently published study[25] reported that, using similar sequencing techniques, less than 10% of healthy infants carried *Enterobacteria* until the sixth week of life when a bloom occurred. Our study went further as we detected specific OTUs that increased in NEC infants during the week prior to diagnosis. Most of these OTUs grouped to the *Enterobacteriaceae*, and represented both known and unknown species. An OTU matching *S. epidermidis*, a common skin commensal, was also exclusively detected in infants with NEC.

We could not confirm a report by others that suggested a contribution of clostridia species and their toxins to NEC.[26] We did not observe an association of NEC risk with the presence of *Citrobacter* like sequences or the frequency of *Enterococc*usas suggested in preliminary studies.[12] However, in the current study we have sufficient power for detecting differences between groups in any OUT's that contributed at least 0.5% to the overall microbiota in the majority of samples from cases or controls.

Our observations regarding any of the OUT's that were more frequently detected in cases should be interpreted with caution. We initially compared distributions of more than a thousand OUT's. Even after removal of rare OUT's we retained many hundreds of OUT's in our analysis. Due to the exploratory character of our study we did not correct for multiple analyses, although we did lower the significance level to p<0.01. Thus, we cannot exclude the possibility that some of our findings did occur by chance. Furthermore, as we studied microbiota development only in preterm infants born in Florida, our findings cannot necessarily be generalized to other populations.

Among the many OTUs that we evaluated we did detect an increase in the numbers of a particular OTU that matched closest to the *Enterobacteriaceae*. When we searched Genbank for the closest match using a blast search, we did not detect any sequence that matched this OTU by more than 97%. This is an intriguing observation because the *Enterobacteriaceae* family is particularly well-studied and contains many established enteric pathogens that harbor a variety of known virulence factors. Our finding is consistent with the hypothesis that a novel pathogen might be an important etiologic factor in at least a subset of infants with NEC. One line of evidence that provides support for the notion that this organism belongs to a class of microbes that has special pathogenic characteristics involves the lipid A moiety of the cell wall LPS, which is known to differ between organisms. Animals use the TLR4 receptor mechanism to recognize the lipid A moiety of LPS. LPS recognition and pro-inflammatory signaling by TLR4 occurs only when lipid A has a certain structure; it contains both phosphates and is a hexaacyl.[27] In the *Bacteroidetes* that sometimes dominate the gut microbiota in adults, the LPS is pentacylated, making it a less efficient activator of the TLR4 mechanism leading to decreased immunogenicity.[28] However in *Proteobacteria*, the lipid A is hexacylated, making it a potent agonist for the TLR4 system and thus more immunogenic. During times of health, the immunogenic properties of the LPS of *Proteobacteria* are muted by the overwhelming predominance of *Bacteroidetes* and *Firmicutes*. However, during inflammation (as occurs in diseases such as NEC), there is a relative decrease in the presence of these less immunogenic bacteria and a relative increase in *Proteobacteria*, as shown by our results.

In addition to specific OTUs that appeared to be associated with NEC we also detected a bloom in *Proteobacteria* unique to the NEC infants. Our observations require confirmation in further studies and with different methodologies. Such studies are currently ongoing at our institution and elsewhere as part of the Human Microbiome Project. We are hopeful that a better understanding of NEC etiology from such studies might be rapidly translated into better diagnostic tools and prophylactic interventions that will meaningfully reduce the incidence and severity of NEC in preterm infants.

## References

[1] Neu J, Walker WA (2011). Necrotizing enterocolitis.. N Engl J Med.

[2] Claud EC, Walker WA (2001). Hypothesis: inappropriate colonization of the premature intestine can cause neonatal necrotizing enterocolitis.. FASEB J.

[3] Morowitz MJ, Poroyko V, Caplan M, Alverdy J, Liu DC (2010). Redefining the role of intestinal microbes in the pathogenesis of necrotizing enterocolitis.. Pediatrics.

[4] Schwiertz A, Gruhl B, Lobnitz M, Michel P, Radke M (2003). Development of the intestinal bacterial composition in hospitalized preterm infants in comparison with breast-fed, full-term infants.. Pediatr Res.

[5] Turnbaugh PJ, Ley RE, Hamady M, Fraser-Liggett CM, Knight R (2007). The human microbiome project.. Nature.

[6] Riesenfeld CS, Schloss PD, Handelsman J (2004). Metagenomics: genomic analysis of microbial communities.. Annu Rev Genet.

[7] Frank DN, Pace NR (2008). Gastrointestinal microbiology enters the metagenomics era.. Curr Opin Gastroenterol.

[8] Hattori M, Taylor TD (2009). The human intestinal microbiome: a new frontier of human biology.. DNA Res.

[9] Palmer C, Bik EM, Digiulio DB, Relman DA, Brown PO (2007). Development of the Human Infant Intestinal Microbiota.. PLoS Biol.

[10] Deshpande G, Rao S, Patole S, Bulsara M (2010). Updated meta-analysis of probiotics for preventing necrotizing enterocolitis in preterm neonates.. Pediatrics.

[11] Wang Y, Hoenig JD, Malin KJ, Qamar S, Petrof EO (2009). 16S rRNA gene-based analysis of fecal microbiota from preterm infants with and without necrotizing enterocolitis.. ISME J.

[12] Mshvildadze M, Neu J, Shuster J, Theriaque D, Li N (2010). Intestinal microbial ecology in premature infants assessed with non-culture-based techniques.. J Pediatr.

[13] Walsh MC, Kliegman RM (1986). Necrotizing enterocolitis: treatment based on staging criteria.. Pediatr Clin North Am.

[14] Bell MJ, Ternberg JL, Feigin RD, Keating JP, Marshall R (1978). Neonatal necrotizing enterocolitis. Therapeutic decisions based upon clinical staging.. Ann Surg.

[15] Mai V, Greenwald B, Morris JG, Raufman JP, Stine OC (2006). Effect of bowel preparation and colonoscopy on post-procedure intestinal microbiota composition.. Gut.

[16] Hamady M, Walker JJ, Harris JK, Gold NJ, Knight R (2008). Error-correcting barcoded primers for pyrosequencing hundreds of samples in multiplex.. Nat Methods.

[17] Sun Y, Cai Y, Liu L, Yu F, Farrell ML (2009). ESPRIT: estimating species richness using large collections of 16S rRNA pyrosequences.. Nucleic Acids Res.

[18] Caporaso JG, Kuczynski J, Stombaugh J, Bittinger K, Bushman FD (2010). QIIME allows analysis of high-throughput community sequencing data.. Nat Methods.

[19] Lozupone C, Hamady M, Knight R (2006). UniFrac--an online tool for comparing microbial community diversity in a phylogenetic context.. BMC Bioinformatics.

[20] O'Hara AM, Shanahan F (2006). The gut flora as a forgotten organ.. EMBO Rep.

[21] Dethlefsen L, Eckburg PB, Bik EM, Relman DA (2006). Assembly of the human intestinal microbiota.. Trends Ecol Evol.

[22] Caicedo RA, Schanler RJ, Li N, Neu J (2005). The developing intestinal ecosystem: implications for the neonate.. Pediatr Res.

[23] Mshvildadze M, Neu J, Mai V (2008). Intestinal microbiota development in the premature neonate: establishment of a lasting commensal relationship?. Nutr Rev.

[24] Sun Y, Cai Y, Mai V, Farmerie W, Yu F (2010). Advanced computational algorithms for microbial community analysis using massive 16S rRNA sequence data.. Nucleic Acids Res.

[25] Jacquot A, Neveu D, Aujoulat F, Mercier G, Marchandin H (2011). Dynamics and clinical evolution of bacterial gut microflora in extremely premature patients.. J Pediatr.

[26] De La Cochetiere MF, Piloquet H, des RC, Darmaun D, Galmiche JP (2004). Early intestinal bacterial colonization and necrotizing enterocolitis in premature infants: the putative role of Clostridium.. Pediatr Res.

[27] Munford RS, Varley AW (2006). Shield as signal: lipopolysaccharides and the evolution of immunity to gram-negative bacteria.. PLoS Pathog.

[28] Coats SR, Do CT, Karimi-Naser LM, Braham PH, Darveau RP (2007). Antagonistic lipopolysaccharides block E. coli lipopolysaccharide function at human TLR4 via interaction with the human MD-2 lipopolysaccharide binding site.. Cell Microbiol.

